# Caregiver burden and distress following the patient's discharge from psychiatric hospital

**DOI:** 10.1192/pb.bp.115.053074

**Published:** 2017-04

**Authors:** Veronica Ranieri, Kevin Madigan, Eric Roche, David McGuinness, Emma Bainbridge, Larkin Feeney, Brian Hallahan, Colm McDonald, Brian O'Donoghue

**Affiliations:** 1 School of Psychology, Trinity College Dublin, Dublin, Ireland; 2 Cluain Mhuire Community Mental Health Service, Blackrock, Co Dublin, Ireland; 3 DETECT, Early Intervention for Psychosis Service, Blackrock, Co Dublin, Ireland; 4 Clinical Science Institute, National University of Ireland, Galway, Ireland; 5 Centre for Youth Mental Health, University of Melbourne, Melbourne, Victoria, Australia; 6 Orygen, the National Centre of Excellence in Youth Mental Health, Melbourne, Victoria, Australia

## Abstract

**Aims and method** Caring for someone with a mental illness is increasingly occurring within the community. As a result, family members who fulfil a caregiving role may experience substantial levels of burden and psychological distress. This study investigates the level of burden and psychological distress reported by caregivers after the patient's admission.

**Results** This study found that the overall level of burden and psychological distress experienced by caregivers did not differ according to the patient's legal status. However, the caregivers of those who were voluntarily admitted supervised the person to a significantly greater extent than the caregivers of those who were involuntarily admitted. Approximately 15% of caregivers revealed high levels of psychological distress.

**Clinical implications** This study may emphasise a need for mental health professionals to examine the circumstances of caregivers, particularly of those caring for patients who are voluntarily admitted, a year after the patient's admission.

One in four families worldwide is affected by mental illness.^1^ With the onset of illness, a family member may assume a caregiver role for their unwell relative, which may result in positive and negative experiences for the caregiver.^2^ While caregivers convey a sense of satisfaction and well-being from their caregiving relationship, they also report feeling burdened.^3^ Such burden may be characterised by both objective difficulties, such as being unable to leave the family home and work, and subjective difficulties, such as psychological distress.^4^

Should a patient become severely unwell, it is often the caregiver who intervenes to initiate emergency psychiatric treatment.^5^ Caregivers can experience significant obstacles in gaining the assistance of a mental healthcare team for their relative^6^ and involuntary admission in particular can be associated with high levels of caregiver burden.^7^ This is important, as the level of burden experienced by caregivers can significantly predict treatment adherence and outcome in the patient.^8^ Furthermore, caregivers who experience high levels of burden reveal an increased incidence of physical and mental health problems and health-related risk behaviours.^8^ However, less is known about the level of burden or psychological distress that caregivers experience in the period following admission. Additionally, the limited research to date has focused on caregivers of involuntarily admitted patients and there is very little known about the caregivers of those admitted voluntarily.

For this reason, we aim to determine the level of burden and psychological distress reported by caregivers approximately 18 months after the patient's admission to an acute mental health unit. We also aim to determine whether clinical (specifically legal status) and demographic factors were associated with the level of burden and psychological distress reported by caregivers.

## Method

### Participants

Participants consisted of caregivers of either involuntarily or voluntarily admitted patients. Caregivers were recruited from two concurrent studies: the Service Users' Perspectives of their Admission (SUPA) study^9^ and the Prospective Evaluation of the Operation and Effects of the Mental Health Act 2001 from the Viewpoints of Service Users and Health Professionals study. The former was conducted in south-east Dublin and North Wicklow and involved both involuntarily and voluntarily admitted patients. The latter was conducted in Galway and Roscommon and included involuntarily admitted individuals and individuals who were brought to hospital under the Irish Mental Health Act, 2001, but were not subsequently involuntarily detained (i.e. they accepted a voluntary admission).

**Fig. 1:**
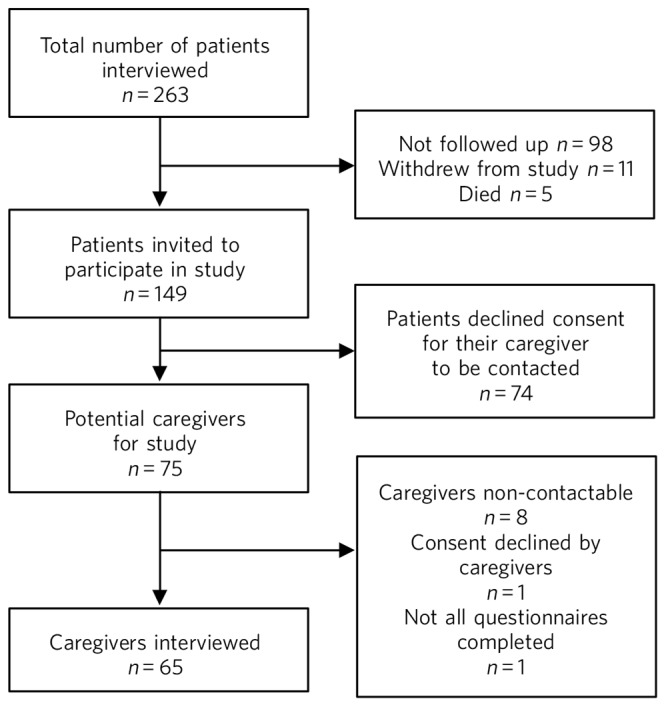
Recruitment of participants, from patient interview to caregiver interview.

As patients were recruited before caregivers in these studies, our inclusion and exclusion criteria for caregivers stemmed from those applied to patients (Fig. 1). Patients were excluded if they could not provide informed consent, had a diagnosis of dementia or had a moderate to severe intellectual disability that rendered them unable to participate in the study. Patients who received a sole diagnosis of a personality disorder or substance misuse were also excluded from participating, as these individuals cannot be admitted involuntarily under the Irish Mental Health Act, 2001. Caregivers who were younger than 18 at the time of interview or who had a moderate to severe intellectual disability that impeded their ability to consent were similarly excluded from participation in the study.

### Informed consent

Informed consent was obtained from all individual participants included in the study. The consent process consisted of three steps. First, patients were asked to consent to their caregiver being contacted and informed of the study approximately 1 year after their discharge. Second, caregivers were contacted by telephone by a researcher who introduced the study and arranged a time of interview. Finally, informed consent was sought from caregivers at interview.

### Psychometric instruments

The Involvement Evaluation Questionnaire (IEQ) was employed as a measure of caregiver burden and caregiving.^10^ It consisted of 27 core items divided into four sections: urging, supervision, tension and worrying. Scores on the IEQ ranged from 0 to 108, with larger figures representing a higher level of caregiver burden. The IEQ is a reliable instrument, with Cronbach's alpha 0.74–0.85 for each subscale and 0.90 for the total score.^11^ The IEQ also included a short, 12-item General Health Questionnaire (GHQ-12) that measured psychological distress. Scores on the GHQ-12 ranged from 0 to 12, with higher scores indicating that the caregiver was experiencing emotional difficulties.^12^ The GHQ-12 is a reliable measure of psychological distress with an alpha coefficient of 0.87.^13^ Finally, diagnostic and clinical information pertaining to the patient was taken from a Structured Clinical Interview for DSM-IV-TR Axis I Disorders (SCID).^14^

### Setting

The study included caregivers of individuals admitted to an acute mental health unit in one of five hospitals in Ireland that covered a combined urban and rural catchment population of over 590 000 individuals.

### Ethical approval

The study received ethical approval from the governing ethical committees in all of the study sites: St John of God Hospitaller Order Provincial Ethics Committee, Newcastle Hospital Ethics Committee and University Hospital Galway Ethics Committee.

### Statistical analysis

All data were entered into a Microsoft Access database and analysed using SPSS Version 22 for Mac. As the data were not normally distributed, multiple Mann–Whitney U and Kruskal–Wallis tests were used to determine whether burden and psychological distress scores differed between caregivers of involuntarily and voluntarily admitted individuals, and according to clinical and demographic factors. Effect sizes (*r*) were used to measure the magnitude of differences between scores. A small, moderate or large effect size corresponded with values equal to or less than 0.10, 0.30 and 0.50, respectively.

## Results

### Demographic and clinical characteristics

Sixty-five caregivers participated in the study; 42 (65%) were female. The mean age of caregivers was 54 years (s.d. = 15). The majority of caregivers were married (*n* = 49, 75%), 5 (8%) were single, a further 5 (8%) were divorced and the remaining 6 (9%) were widowed. Most caregivers were the person's parents (*n* = 37, 57%), 11 (17%) were spouses or partners, 8 (12%) were siblings, 6 (9%) were children and 3 (5%) were other relatives. The mean length of time between caregiver and patient interviews at baseline was 584 days (s.d. = 165). The median duration of the index admission was 34 days (interquartile range (IQR) 17.5–50) and 24 (36.9%) patients were readmitted within 1 year of the index admission.

Forty-six (71%) individuals were involuntarily admitted and 19 (29%) were voluntarily admitted. The majority were male (*n* = 33, 51%). The mean age of patients was 39 years (s.d. = 12). The majority of patients were single (*n* = 41, 63%), 16 (25%) were married and the remaining 8 (12%) were divorced.

Caregiver characteristics across legal status are given in Table 1.

**Table 1:** Comparison of caregiver characteristics across legal status

Characteristic	All caregivers	Caregivers of involuntarily admitted patients	Caregivers of voluntarily admitted patients	Statistical test	*P*
Age, years: median (IQR)	54 (43–67)	54 (43–66)	54 (39–68)	*U* = 340	0.94

Gender, *n* (%)					
Male	23 (35)	14 (30)	9 (47)	χ^2^ = 1.7 d.f. = 1	0.19
Female	42 (65)	32 (70)	10 (53)		

Marital status, *n* (%)					
In relationship	49 (75)	34 (74)	15 (79)	χ^2^ = 0.2 d.f. = 1	0.67
Not in relationship	16 (25)	12 (26)	4 (21)		

Education, *n* (%)					
Primary/secondary	16 (30)	12 (33)	2 (13)	χ^2^ = 2.8 d.f. = 1	0.09
Tertiary	37 (70)	24 (67)	13 (87)		

Household, *n* (%)					
Living together	37 (57)	23 (50)	14 (74)	χ^2^ = 3.1 d.f. = 1	0.08
Living separately	28 (43)	23 (50)	5 (26)		

Relationship, *n* (%)					
Parent	34 (71)	25 (78)	9 (56)	χ^2^ = 2.5 d.f. = 1	0.12
Partner	14 (29)	7 (22)	7 (44)		

IQR, interquartile range.

### Caregiver burden

The median level of burden in the sample was 13.00 (IQR 6.00–22.00). The median level of burden in caregivers of involuntarily admitted patients was 11.50 (IQR 6.25–20.75) and in caregivers of those voluntarily admitted it was 18.00 (IQR 5.00–34.00). Caregivers' overall scores of burden did not significantly differ (*U* = 328, *P* = 0.18, *r* = 0.17). However, caregivers of voluntarily admitted patients supervised the person to a greater extent than caregivers of involuntarily admitted patients (median IEQ supervision scores 2 *v.* 0, *U* = 258, *P* < 0.001, *r* = 0.38). The particular items of the subscale of supervision on which caregivers of voluntarily admitted individuals scored higher were ensuring that the person had enough sleep (*P* = 0.02) and that they did not drink too much alcohol (*P* = 0.05).

### Factors associated with caregiver burden

There was no significant association between the level of caregiver burden and the caregiver's gender, patient's gender, diagnosis, level of functioning, relationship to caregiver or living in the same household as the patient (Table 2).

**Table 2:** Involvement Evaluation Questionnaire (IEQ) and General Health Questionnaire (GHQ-12) scores according to demographic and clinical characteristics

	IEQ					
	Total burden	Urging	Supervision	Tension	Worrying	GHQ-12

	Median (IQR)					
Caregiver gender						
Male	9 (5–22)	3 (1–8)	0 (0–2)	2 (0–5)	3 (1–7)	1 (0–2)
Female	14 (8–23)	4 (1–7)	0 (0–1)	3 (2–6)	6 (3–8)	1 (0–3)

Patient gender						
Male	10 (5–23)	4 (1–9)	0 (0–2)	2 (1–4)	5 (2–8)	1 (0–2)
Female	14 (8–22)	3 (1–8)	0 (0–2)	4 (2–7)	5 (3–8)	1 (0–3)

Diagnosis						
Affective	11 (7–21)	3 (1–6)	0 (0–2)	3 (1–6)	4 (1–7)	1 (0–2)
Psychotic	14 (8–21)	5 (1–12)	0 (0–3)	3 (0–6)	6 (3–9)	1 (0–3)

GAF score						
Higher functioning	12 (5–23)	3 (1–8)	0 (0–2)	4 (0–7)	5 (1–9)	1 (0–2)
Lower functioning	14 (7–21)	4 (1–9)	0 (0–2)	3 (1–6)	5 (2–8)	1 (0–3)

Relationship of caregiver to patient						
Parent	11 (5–21)	3 (1–6)	0 (0–2)	3 (1–6)	5 (3–8)	1 (0–3)
Partner	21 (7–28)	6 (1–11)	1 (0–2)	4 (2–7)	4 (2–10)	1 (0–2)

Household						
Living together	15 (6–24)*	5 (2–10)*	0 (0–2)	4 (1–6)	6 (2–9)	1 (0–2)
Living separately	9 (6–20)	2 (1–5)	2 (1–6)	2 (1–6)	4 (1–7)	1 (0–3)

GAF, Global Assessment of Functioning; IQR, interquartile range.

* *P* ⩾ 0.05. On applying a Bonferroni correction, no variable reached significance.

### Psychological distress

Nine caregivers (15%) reported high levels of distress (defined as a score of ⩾4 on GHQ-12). The median score of psychological distress was 1.00 (IQR 0.00–2.50): 1.00 (IQR 0.00–3.00) in caregivers of involuntarily admitted patients and 0.00 (IQR 0.00–1.00) in caregivers of voluntarily admitted patients. Caregivers of involuntarily or voluntarily admitted patients did not significantly differ in their levels of psychological distress (*U* = 302, *P* = 0.19).

### Factors associated with psychological distress

No significant association was found between the caregiver's level of psychological distress and the caregiver's gender, patient's gender, diagnosis, level of functioning, relationship to caregiver or whether they lived in the same household as the patient (Table 2).

### *Post hoc* analysis

We hypothesised that subsequent admissions from the index admission could affect the burden and psychological distress of caregivers and therefore further analysis examining this was performed. Caregiver burden was higher when the patients had been readmitted (16.0 *v.* 9.5, *U* = 281.5, *P* = 0.04) and there was a trend for a higher level of distress (1.0 *v.* 0.0, *U* = 277, *P* = 0.06). Legal status for the index admission was not associated with readmission (χ^2^ = 0.28, *n* = 61, *P* = 0.60).

## Discussion

### Summary of findings

The findings indicated that overall levels of burden and psychological distress did not differ between caregivers of involuntarily and voluntarily admitted patients at approximately 18 months after discharge from an acute mental health unit. However, caregivers of voluntarily admitted patients engaged in significantly higher levels of supervision than caregivers of those involuntarily admitted. Such supervision focused on, for example, ensuring that the patient slept sufficiently. *Post hoc* analysis also suggested that caregiver burden is associated with readmission to hospital.

### Comparison with previous research

To our knowledge, no study to date has examined whether differences in the patient's legal status at admission accounted for differences in caregiver burden (objective and subjective) at more than 1 year follow-up. Our findings, however, support those of Boydell *et al*,^7^ who also emphasised that overall burden was not linked to involuntary admission in caregivers of patients with first-episode psychosis. Our scores of burden and psychological distress are substantially lower than those reported by other authors.^11^ Thus, the similarity in scores between caregivers in this sample may be due to a reduction in overall caregiver burden following the person's admission to hospital regardless of legal status.^15^

### Implications

The finding that caregivers of voluntarily admitted individuals supervised the person to a greater extent is interesting and warrants discussion. It is possible that this additional supervision is a positive experience and that it results in an earlier detection of warning signs and prevents potential relapses from progressing to an involuntary admission. This finding highlights the need for caregivers of both voluntarily and involuntarily admitted individuals to receive support and psychoeducation. Interestingly, the *post hoc* analysis suggests that it may be the frequency of admissions that results in higher burden, as opposed to the legal status of the admissions. Furthermore, a longitudinal replication of this study assessing burden at various time points may provide us with a clearer picture of the caregiver's experience of burden.

### Strengths and limitations

The study encompassed a number of strengths and limitations. Our sample included caregivers of involuntarily and voluntarily admitted individuals from both rural and urban geographical locations. Another strength of the study was that the caregivers did not self-select to the study. Nonetheless, our process of consent may have introduced bias, as those who consented for their caregiver to be interviewed may have had closer family relationships. Additionally, there was a significant gap between the index admission and the caregiver interviews, which introduces a number of potential confounders, such as readmission to hospital.
